# Determination of Bongkrekic Acid and Iso-Bongkrekic Acid in Red Yeast Rice and Its Related Products by Liquid Chromatography-Tandem Mass Spectrometry

**DOI:** 10.3390/toxins18100407

**Published:** 2026-09-22

**Authors:** Yuan Guo, Yuxin An, Jiaojiao Xu, Zengxuan Cai, Meijia Xu, Xiaomin Xu

**Affiliations:** 1 NHC Key Laboratory of Food Safety Risk Assessment, China National Center for Food Safety Risk Assessment, Beijing 100026, China; guoyuan@cfsa.net.cn; 2 NHC Specialty Laboratory of Food Safety Risk Assessment and Standard Development, Zhejiang Provincial Center for Disease Control and Prevention, Hangzhou 310051, China; yxan@cdc.zj.cn (Y.A.); jjxu@cdc.zj.cn (J.X.); zxcai@cdc.zj.cn (Z.C.); mjxu@cdc.zj.cn (M.X.); 3 Zhejiang Key Laboratory of Public Health Detection and Pathogenesis Research, Zhejiang Provincial Center for Disease Control and Prevention, Hangzhou 310051, China

**Keywords:** bongkrekic acid, iso-bongkrekic acid, red yeast rice and its related products, anion-exchange cartridge, LC-MS/MS

## Abstract

Bongkrekic acid (BA) is a metabolite that is lethal to humans and is produced by *Burkholderia gladioli* pathovar *cocovenenans* (BGC) containing the bon gene cluster. An analytical method for BA and its stereoisomer, iso-bongkrekic acid (IBA), was established for deeply fermented rice products, such as red yeast rice and its related products, by liquid chromatography–tandem mass spectrometry (LC-MS/MS). BA and IBA in samples were extracted with 80% methanol/water containing 1% ammonia by ultrasonic assistance, cleaned by solid-phase extraction (SPE) with an anion-exchange cartridge, separated with an XBridge^TM^ BEH C_18_ chromatographic column (150 mm × 3.0 mm, 2.5 μm), measured by MS/MS, and quantified using an isotope-labeled internal standard. The influencing factors such as extraction solvents, ultrasonic extraction time and loading volume during SPE clean-up were optimized to improve the recoveries of BA and IBA. Also, the extraction efficiency between the simulated quality control and directly spiked samples, the matrix effects with the use of different chromatographic columns, the impact of co-eluting interferences under different mobile phase compositions and the conversion of BA to IBA are discussed. The linear range of BA and IBA was 0.1–50 ng/mL (equivalent to 1–500 μg/kg in the sample), with the correlation coefficient exceeding 0.999. The limits of detection and quantification were 0.3–0.5 μg/kg and 1.0–1.5 μg/kg, respectively. The average recoveries of the analytes were 88.4–107% with relative standard deviations (RSDs, *n* = 6) between 2.9% and 11.8% in red yeast rice and its related products under three spiking levels. This optimized method was applied to study the contamination of BA and IBA in commercially available samples. This study can provide technical support for the supervision and risk assessment of BA and IBA in red yeast rice and its related products.

## 1. Introduction

Bongkrekic acid (BA) is a toxic metabolite produced by *Burkholderia gladioli* pathovar *cocovenenans* (BGC) containing the bon gene cluster [1,2,3]. BA is an efficient and highly selective inhibitor of the mitochondrial ATP/ADP carrier. It mainly acts on the inner mitochondrial membrane and induces mitochondrial dysfunction by inhibiting adenine nucleotide translocase [4]. The oral LD_50_ of BA in mice is 3.16 mg/kg [5], and the minimum lethal dose is only 1–1.5 mg for a human adult [6]. BA is one of the most lethal biotoxins in foodborne poisoning incidents [7,8,9,10].

Typical foods easily contaminated with BA include fermented rice and corn flour products, coconut, *Tremella fuciformis* and black fungus in Asia, Africa, and North America [1,4,7,8,10,11]. In 2020, a poisoning event was reported with 100% mortality among nine patients in Heilongjiang Province, China, due to the intake of BA-contaminated sour soup, a homemade fermented corn product [9]. BA poisoning cases related to unfermented rice products have been reported since 2018 in Guangdong and Zhejiang Provinces, China [8]. In 2024, a BA poisoning event was reported with a mortality of 18.2% in 33 patients caused by an unfermented rice product in Taiwan, China [12,13]. The maximum limit for BA is set at 0.25 mg/kg in *Tremella fuciformis* in China [14]. No limit for BA has been set for the other foods.

Red yeast rice is a deeply fermented rice product. It is traditionally used in Asia as a dietary supplement for cholesterol management [15,16]. It is often combined with other foods such as natto (a fermented soybean product) to make red yeast rice-related health foods in China. Furthermore, it is used to extract red pigment (called *monascus* red pigment) as a coloring agent for food additives. From late 2023 to early 2024, poisoning events involving renal dysfunction related to the specific Beni-koji (red yeast rice) tablets were reported in Japan [17,18]. It proposes a new challenge to the food safety risk of biotoxin contamination in such fermented food products. Fermented rice products are one of the main carriers and culture media for BGC [9]. BA contamination in red yeast rice and its related products is possible. However, few previous studies have reported BA and IBA contamination in this matrix.

BA is a long-chain fatty acid containing three carboxyl groups (Figure 1). Analytical methods for BA in foods include the immunoassay [11,19,20], high-performance liquid chromatography (HPLC) [21,22], and liquid chromatography–tandem mass spectrometry (LC-MS/MS) [11,13,22,23,24,25,26,27,28]. Fast screening results for BA can be obtained without expensive instruments by using immunoassay methods. Accurate qualitative and quantitative results of BA in foods can be obtained by HPLC or LC-MS/MS [11]. Moreover, the LC-MS/MS method is the most effective solution for the determination of BA in foods [11]. BA can be converted into its stereoisomer, iso-bongkrekic acid (IBA, Figure 1), during food processing and storage [24]. BA and IBA can be simultaneously detected by LC-MS/MS [23,24].

The extraction and clean-up methods for BA in foods depend on the complexity of the matrix. Direct extraction with acetic acid and acetonitrile [24] or ammonia/methanol solution was used to detect BA in *Tremella fuciformis* or rice-derived products [26]. For foods with relatively complex matrix interference, methanol/water [23] or acetonitrile/water [28] extraction plus HLB clean-up, acetic acid/acetonitrile extraction plus solid-phase extraction (SPE) clean-up with a weak anion-exchange cartridge [24], or alkaline methanol/water extraction plus SPE clean-up with a strong anion-exchange cartridge [22,27] was required. The color of red yeast rice is related to the degree of fermentation. To obtain a darker color, deep fermentation is required. Red-yeast health care foods are a mixture of red yeast rice and high-protein foods, and the matrix is more complex. The above methods were used to detect BA in red yeast rice and its related products while the matrix suppression effects were severe or the recoveries were unsatisfactory. This article describes the development of an analytical method with alkaline methanol/water extraction combined with anion-exchange SPE (PAX cartridge) clean-up for BA and IBA in red yeast rice and its related products using LC-MS/MS.

## 2. Results and Discussion

### 2.1. Selection of Extraction Solvent

The recoveries of BA and IBA extracted with 100% to 70% methanol/water containing 1% ammonia in spiked red yeast rice (SP), quality control (QC) samples of red yeast rice (QC-A), the natto red yeast health care product (QC-B) and *monascus* red pigment (QC-C) are shown in Figure 2. For the spiked sample (SP), the recoveries of BA and IBA were more than 90% when extracted with 100% to 70% methanol/water containing 1% ammonia. However, the recoveries of BA and IBA were less than 70% for the QC samples when no water was added to the extraction solvent (100% methanol containing 1% ammonia). The recoveries increased when water was added in the extraction solvent and the results exceeded 90% with an extraction solvent of 80% methanol/water containing 1% of ammonia. The presence of interfering substances with anion-exchange adsorption capacity would have increased in the extract if more water was added. This would affect the recoveries of the analytes during SPE clean-up.

BA and IBA were encapsulated within the dried sample matrix of red yeast rice and its related products. Methanol could not penetrate into the interior of the dried matrix without water-assisted extraction, and low recoveries were obtained. For the spiked sample, the analytes remained on the surface of the matrix and the recoveries were more than 90% without water-assisted extraction. Therefore, erroneous conclusions regarding extraction efficiency would have been drawn for the dried samples if the direct spiking method was used. Based on the extraction efficiency of the QCs, the optimized extraction solvent was 80% methanol/water containing 1% ammonia.

The solvents with 80% and 70% methanol/water (each containing 1% ammonia) were used to extract BA and IBA, respectively, in the naturally contaminated samples of red yeast rice (17.7 μg/kg of BA and 12.4 μg/kg of IBA), the red yeast health care product (9.1 μg/kg of BA and 7.6 μg/kg of IBA) and *monascus* red pigment (34.0 μg/kg of BA and 39.1 μg/kg of IBA). The relative deviations of the contents were below 10%. Therefore, the optimized extraction solvent was suitable for the determination of the analytes in the naturally contaminated samples.

### 2.2. Selection of Ultrasonic Extraction Time

The recoveries of BA and IBA during the ultrasonic extraction time of 20 min–50 min in SP, QC-A, QC-B and QC-C are shown in Figure 3. For SP, the recoveries of BA and IBA were more than 90% with the use of the ultrasonic extraction time of 20 min–50 min. However, the recoveries of BA and IBA were less than 80% in QC-A and QC-B under an ultrasonic extraction time of 20 min. The recoveries of the analytes in all the QC samples were improved as the ultrasonic extraction time increased and the results were more than 90% with an ultrasonic extraction time of 40 min.

The ultrasonic extraction durations of 40 min and 50 min were used to extract BA and IBA, respectively, in the naturally contaminated samples of red yeast rice (17.7 μg/kg of BA and 12.4 μg/kg of IBA), the red yeast health care product (9.1 μg/kg of BA and 7.6 μg/kg of IBA) and *monascus* red pigment (34.0 μg/kg of BA and 39.1 μg/kg of IBA). The relative deviations of the contents were below 10%. Therefore, the optimized ultrasonic extraction time was suitable for the determination of the analytes in naturally contaminated samples as well.

### 2.3. Optimization of Loading Volume for SPE Clean-Up

The loading volume (0.2 mL–1.0 mL) of the sample extract for QC-A, QC-B and QC-C was optimized for SPE clean-up with the PAX cartridge. The internal standard (IS) was spiked after clean-up to evaluate SPE recovery. The results are shown in Figure 4. The recoveries of the analytes were 63.4–85.5% in QC-A and QC-B and 31.5–34.7% in QC-C with a loading volume of 1.0 mL. The recoveries were above 90% in QC-A and QC-B and 72.1–79.1% in QC-C with a loading volume of 0.5 mL. The recoveries were more than 80% in QC-C with a loading volume of 0.2 mL. The reported anion-exchange SPE loading volume was 5 mL for the normal food matrix [11,22]. However, there are numerous interfering substances with carboxyl groups that exhibit anion-exchange function in deeply fermented sample matrices like red yeast rice. A loading volume of 1.0 mL may cause overloading of the SPE cartridge and reduce the recoveries of the analytes. Such interfering substances are more common in the red pigment (QC-C). Based on the recovery results of BA and IBA in the three studied matrices (Figure 4), the preferable SPE loading volume was 0.5 mL. Although the recoveries in QC-C failed to reach 80% during SPE clean-up, the loss can be corrected by IS calibration.

### 2.4. Selection of the Mobile Phase

The mobile phase with 0.1% formic acid (aqueous phase) and acetonitrile (organic phase) was used for the separation of BA by C_18_ column in foods except for red yeast rice products [22,23,26]. Remarkable co-eluting matrix interfering peaks were found for BA in red yeast rice samples with the use of this mobile phase (Figure 5(1a)). Remarkable matrix suppression effects were found for BA, caused by the co-eluting interference peaks (Figure 5(1b)). It was difficult to obtain accurate qualitative and quantitative results. In this study, the addition of ammonium formate in the water phase was found to improve the separation of BA from the interfering substances. Good results were observed as the concentration of ammonium formate increased from 0.5 mmol/L (Figure 5(2a,2b)) to 1 mmol/L (Figure 5(3a,3b)). However, the instrument signals of the analytes were found to decrease with the addition of ammonium formate. Therefore, 0.1% of formic acid and 1 mmol/L of ammonium formate were used as the preferred aqueous phase for the separation of BA and IBA in red yeast rice and its related products in this study.

### 2.5. Matrix Effects and the Selection of the Chromatographic Column

The matrix effects of BA and IBA in red yeast rice, the natto red yeast health care product and *monascus* red pigment separated by C_18_ columns with the lengths of 100 mm and 150 mm, respectively, are shown in Figure 6. To improve the detection efficiency, BA was separated by 100 mm of the C_18_ column from foods except for red yeast rice products [11,22,23]. In this study, the matrix effects of BA and IBA in the above three matrices were 47.2–70.3% when separated by 100 mm of the C_18_ column. Strong matrix effects were found since half of the instrument signals were suppressed in the *monascus* red pigment. The matrix effects of BA and IBA in the above three matrices were effectively improved to 78.8–88.6% when separated by 150 mm of the C_18_ column.

### 2.6. Method Validation

#### 2.6.1. Linear Range, Limits of Detection and Quantification

With IS calibration, good linear correlations were found for BA and IBA within the concentration range of 0.1–50 ng/mL (equivalent to 1–500 μg/kg in the sample). The linear regression equations were y = 1.27419x + 0.018433 and y = 1.20691x + 0.017306 with the correlation coefficients of 0.9990 and 0.9993 for BA and IBA, respectively.

The limit of detection (LOD) and the limit of quantification (LOQ) are defined as the concentrations corresponding to instrumental signal-to-noise ratios of 3 and 10, respectively, for the analytes in the matrix. The LOD and LOQ were 0.5 μg/kg and 1.5 μg/kg, respectively, for the analytes in the *monascus* red pigment. The results were 0.3 μg/kg and 1.0 μg/kg, respectively, for the analytes in the other sample matrices.

#### 2.6.2. Accuracy and Precision

The recovery and relative standard deviation (RSD, *n* = 6) were used to evaluate the accuracy and precision of the method, respectively. The intra-day accuracy and precision were measured in the spiked samples with the concentrations of the analytes at 1 μg/kg (1.5 μg/kg for *monascus* red pigment), 20 μg/kg and 200 μg/kg, respectively. The results are listed in Table 1. The average recoveries of the analytes were 92.2–107%, 92.8–97.1% and 88.4–104% with RSDs of 4.0–8.9%, 2.9–6.4% and 4.6–11.8% in red yeast rice, the natto red yeast health care product and *monascus* red pigment, respectively.

The inter-day accuracy and precision were measured by testing on three consecutive days (*n* = 3) using the QC samples of QC-A, QC-B and QC-C at 20 μg/kg for BA and IBA. The results are listed in Table 2. The accuracy values for the analytes were 91.1–97.9% with RSDs of 6.3–9.2% in red yeast rice, the natto red yeast health care product and *monascus* red pigment.

#### 2.6.3. Carry-Over Evaluation and Dilution Effects

Carry-over effects were evaluated by measuring the analytes in the pure solvent injected after the extract of spiked samples at a high concentration (1000 μg/kg) in the typical sample matrices of red yeast rice, natto red yeast health care product and *monascus* red pigment, respectively. No cross contamination was observed since no peaks of the analytes were found in the pure solvent measured by LC-MS/MS.

The recoveries and RSDs (*n* = 6) of BA and IBA are listed in Table 1 for the three typical samples spiked at 1000 μg/kg and subjected to a 5-fold dilution. The average recoveries of the analytes were 92.2–107% with RSDs of 4.0–8.9%. Acceptable results were obtained after dilution for the samples with concentrations exceeding the upper limit of the linear range (500 μg/kg).

### 2.7. Method Application

The optimized analytical method was applied to detect BA and IBA in red yeast rice and its related products. The samples included 80 red yeast rice products, 26 red yeast health care products and 29 *monascus* red pigment samples (Table 3). The detection rates of BA were 22.5%, 19.2% and 20.7% with the maximum concentrations of 17.7 μg/kg, 9.1 μg/kg and 1320 μg/kg, respectively. The detection rates of IBA were 16.3%, 15.4% and 20.7% with maximum concentrations of 12.4 μg/kg, 7.6 μg/kg and 1647 μg/kg, respectively. The maximum concentration of BA in the *monascus* red pigment was more than 1000 μg/kg, which requires attention. The contamination levels of BA and IBA in the other samples were relatively low.

IBA is converted from BA. This conversion is enhanced as time increases during food processing and storage [24]. The production time and storage time of the red yeast rice products are relatively long, and the conversion of BA to IBA cannot be avoided. The *monascus* red pigment samples were processed products extracted from red yeast rice. The concentrations of IBA were higher than those of BA and the detection rates were the same. These results provide evidence for the above conversion.

To accurately evaluate the contamination level of BA, it is necessary to simultaneously detect the contents of BA and IBA in food. At the same time, it is necessary to evaluate the conversion of BA to IBA in the standard solution during testing. It was found that no signal of IBA was observed in the newly purchased BA standard solution, and no chromatographic peak of BA was observed in IBA solution. However, after being stored for more than 6 months, a clear IBA chromatographic peak can be observed in BA standard solution, while no BA peak is observed in IBA solution. The sample extract spiked with only BA or IBA was put in the autosampler for stability testing. No interconversion between BA and IBA was observed after storage for 48 h. As shown in Figure 1, the conversion from BA to IBA may be related to the formation of an intramolecular H-bond for IBA. Due to intramolecular hydrogen bonding, the structure of IBA is more stable than that of BA.

The conversion of BA to IBA in the standard solution will affect the quantitative results. To accurately determine the contents of BA and IBA in the sample, the standard solutions should be stored separately as single-analyte standard solutions. The purity of BA should be checked before mixing. If the IBA signal in the BA standard solution exceeds 5% of the BA signal, a fresh standard solution should be prepared and used.

## 3. Conclusions

The established analytical method for BA and IBA in red yeast rice and its related products involving SPE clean-up and LC-MS/MS measurement is simple and rapid. The optimized chromatographic separation conditions can effectively improve the interference from the co-eluting substances in the matrix and ensure accurate quantitative results.

The experimental results indicate that for dried matrices, the effectiveness of extraction solvents and methods cannot be evaluated by the direct spiking procedure. Instead, simulated QC samples or positive samples must be employed for method optimization. For samples with severe interference, the loading volume of the sample extract should be assessed when using ion exchange cartridge for clean-up.

The conversion of BA to IBA occurs during sample processing and storage, as well as during standard solution storage. To obtain accurate quantitative results, it is necessary to prevent and assess this conversion during analysis.

## 4. Materials and Methods

### 4.1. Materials and Reagents

All reagents and solvents were of analytical grade unless specified.

BA (100 mg/L, ≥98%), IBA (100 mg/L, ≥98%) and the IS of ^13^C_28_-BA (10 mg/L, ≥98%) were obtained from Alta Scientific Co., Ltd. (Tianjin, China). HPLC-grade methanol and acetonitrile were provided by TEDIA (Fairfield, OH, USA). Ammonium formate was obtained from Sigma-Aldrich (St. Louis, MO, USA). Formic acid (MS grade) and ammonia solution (>25%) were provided by Thermo Fisher Scientific (Shanghai, China). The anion-exchange SPE cartridge (PAX, 60 mg/3 mL) was supported by Agela Technologies (Tianjin, China). Red yeast rice and its related products including red yeast health care products and *monascus* red pigment samples were purchased from local markets or online shopping platforms.

The mixed standard spiking solutions of BA and IBA (10,000 μg/L, 1000 μg/L and 10 μg/L) were prepared by diluting the stock solutions stepwise with methanol. The IS spiking solution of ^13^C_28_-BA (500 μg/L) was prepared by diluting the IS stock solution with methanol. A series of standard solutions with concentrations of 0.1–50 ng/mL (equivalent to 1–500 μg/kg in the sample) was prepared using 50% (*v*/*v*) acetonitrile/water. To each milliliter of the standard solution, 10 μL (5 ng) of the IS spiking solution was added.

### 4.2. Equipment

A liquid chromatography–triple quadrupole mass spectrometer (LCMS-8060, Shimadzu, Tokyo, Japan) was used for measurement. A Millipore system (Bedford, MA, USA) was used for the preparation of ultrapure water. A high-speed pulverizer (25,000 rpm, Linda Machinery, Wenling, China) was used for milling solid samples. A blender (32,000 rpm, Midea Group, Foshan, China) was used for homogenization of wet samples.

### 4.3. Sample Preparation

Red yeast rice and its related products: All samples were finely milled with a high-speed pulverizer, mixed thoroughly and stored at −20 °C.

QC sample: An aliquot of 200 g BA- and IBA-negative red yeast rice, natto red yeast health care product, or *monascus* red pigment was mixed with 200 mL of water and 0.4 mL of a mixed standard spiking solution of BA and IBA (10,000 μg/L) and then homogenized using a blender. After freeze-drying, the samples were finely milled with a high-speed pulverizer and mixed thoroughly to obtain the QC samples of red yeast rice (QC-A), the natto red yeast health care product (QC-B), and *monascus* red pigment (QC-C), all with a concentration of 20 μg/kg for BA and IBA.

### 4.4. Sample Extraction and Clean-Up

The sample extraction and clean-up procedures were developed based on the Chinese National Food Safety Standard, Determination of Bongkrekic Acid in Food (GB 5009. 189–2023) [22]. An aliquot of 2 g of the homogenized sample was weighed into a 15 mL polypropylene centrifuge tube. After the addition of 200 μL of IS (500 μg/L) and 10 mL of 80% methanol/water containing 1% ammonia, the sample was extracted using ultrasound for 40 min, and then centrifuged at 8000 rpm for 5 min. An aliquot of 0.5 mL of the supernatant was mixed with 2 mL of 80% methanol/water containing 1% ammonia and loaded onto the PAX cartridge, which was conditioned with 3 mL of methanol and 3 mL of water before use. The PAX cartridge was washed with 3 mL of water and 3 mL of methanol after sample loading was completed. The analytes were then eluted from the cartridge with 3 mL of 1% formic acid in methanol. All the eluate was collected and dried at 40 °C under a gentle stream of nitrogen. The residue was dissolved in 1.0 mL of 50% acetonitrile/water by vortex mixing. The solution was filtered using a Teflon membrane (0.2 μm) before LC-MS/MS analysis.

### 4.5. Condition Optimization of the Extraction Solvent

Aliquots of 2 g of each of QC-A, QC-B, QC-C and red yeast rice without BA were weighed into different 15 mL polypropylene centrifuge tubes. The red yeast rice without BA was spiked with 40 μL of a mixed standard solution of BA and IBA (1000 μg/L) to prepare the spiked sample (SP) at a concentration of 20 μg/kg. The spiked sample was mixed and left to stand for 30 min. The QC and SP samples were extracted with 10 mL of 100%, 90%, 80%, 70% methanol/water containing 1% ammonia, respectively. Ten microliters of IS (500 μg/L) was spiked into 0.5 mL of the supernatant after ultrasonic extraction. The other steps were the same as those described in Section 4.4. Three repetitions were performed for each condition.

### 4.6. Condition Optimization of Ultrasonic Extraction Time

The QC and SP samples (prepared as mentioned in Section 4.5) were each mixed with 10 mL of 80% methanol/water containing 1% ammonia. Ultrasonic extraction was performed for 20 min, 30 min, 40 min and 50 min, respectively. Ten microliters of IS (500 μg/L) was spiked into 0.5 mL of the supernatant after ultrasonic extraction. The other steps were the same as those described in Section 4.4. Three repetitions were performed for each condition.

### 4.7. Optimization of the SPE Clean-Up Conditions

Aliquots of 2 g of QC-A, QC-B and QC-C were weighed into different 15 mL polypropylene centrifuge tubes. After ultrasonic extraction with 10 mL of 80% methanol/water containing 1% ammonia for 40 min, 0.2 mL, 0.5 mL and 1.0 mL of the supernatants, respectively, were used for SPE clean-up. Ten microliters of IS (500 μg/L) was spiked into each eluate after SPE clean-up. The other steps were the same as those described in Section 4.4. Three repetitions were performed for each condition.

### 4.8. Matrix Effects

Aliquots of 2 g of each of the BA- and IBA-negative red yeast rice (A), natto red yeast health care product (B) and *monascus* red pigment (C) samples were processed as described in Section 4.4. The resulting residue after SPE and drying was dissolved in the mixed standard spiking solution of BA and IBA (10 μg/L) to obtain the matrix-matched standard solution. Three repetitions were performed for each matrix. Matrix effects were calculated as 100 × (peak area of the analyte in matrix/peak area in pure solvent). The results more or less 100% represent the matrix enhancement or suppression effects [29].

### 4.9. Accuracy and Precision of the Method

Intra- and inter-day accuracy and precision were evaluated in this study.

Aliquots of 2 g of each of the BA- and IBA-negative red yeast rice, natto red yeast health care product and *monascus* red pigment samples were weighed into different 15 mL polypropylene centrifuge tubes. Each sample was spiked at three levels with mixed standard solutions of BA and IBA: 200 μL of 10 μg/L (300 μL was used for *monascus* red pigment), 40 μL of 1000 μg/L, and 200 μL of 1000 μg/L. The samples were allowed to stand for 30 min after mixing. The other steps were the same as those described in Section 4.4. Six replicates were performed for each condition. Recovery and RSD were calculated to evaluate intra-day accuracy and precision, respectively.

Aliquots of 2 g of each of the QC-A, QC-B and QC-C samples were weighed into different 15 mL polypropylene centrifuge tubes. The samples were measured on three consecutive days with 3 replicates for each day. The recovery and RSD were calculated for the evaluation of inter-day accuracy and precision, respectively.

### 4.10. Carry-Over and Dilution Integrity Evaluation

Aliquots of 2 g of each of the BA- and IBA-negative red yeast rice, natto red yeast health care product and *monascus* red pigment samples were weighed into different 15 mL polypropylene centrifuge tubes. Each sample was spiked with 200 μL of a 10,000 μg/L mixed standard solution (equivalent to 1000 μg/kg) and 200 μL of IS spiking solution (2500 μg/L). After mixing, the samples were allowed to stand for 30 min. For the carry-over evaluation, the other steps were the same as those described in Section 4.4. A pure solvent mixture consisting of 50% acetonitrile/water was injected for LC-MS/MS measurement after the spiked sample. For dilution integrity evaluation, only 0.1 mL of the supernatant was used for SPE clean-up (equivalent to a 5-fold dilution). Six replicates were performed for each matrix.

### 4.11. LC-MS/MS Analysis

The chromatographic separation of BA and IBA was performed on an XBridge^TM^ BEH C_18_ column (150 mm × 3.0 mm, 2.5 μm, Waters, Wexford, Ireland) with a flow rate of 0.4 mL/min at a column temperature of 35 °C. The mobile phases were (A) water containing 0.1% formic acid and 1 mmol/L ammonium formate and (B) acetonitrile. The gradient program was as follows: 20% B (0–0.5 min), 20%–90% B (0.5–7.0 min), 90% B (7.0–8.0 min), 90% –20% B (8.0–8.5 min), and 20% B (8.5–11.0 min). The injection volume was 2 μL.

The MS measurement was performed as follows: electrospray ionization in negative mode (ESI^−^); ion source interface voltage: 4.5 kV; nebulizer gas and drying gas: nitrogen, 3.0 L/min and 10 L/min; heating gas: air, 10 L/min; collision gas: argon; desolvent tube, heating block and interface temperatures: 250 °C, 400 °C and 300 °C, respectively; multiple reaction monitoring (MRM) mode, BA and IBA: 485.2 > 441.2 (14 eV, quantitative ion pair), 485.2 > 397.2 (18 eV); IS: 513.2 > 468.2 (14 eV, quantitative ion pair), 513.2 > 423.2 (18 eV).

### 4.12. Data Analysis

Data acquisition and processing were performed using LabSolutions Insight (version V1.5, Shimadzu, Tokyo, Japan). Statistical analysis was performed using GraphPad Prism version 11, Microsoft Excel 2016 and Microsoft PowerPoint 2016.

## Figures and Tables

**Figure 1:**
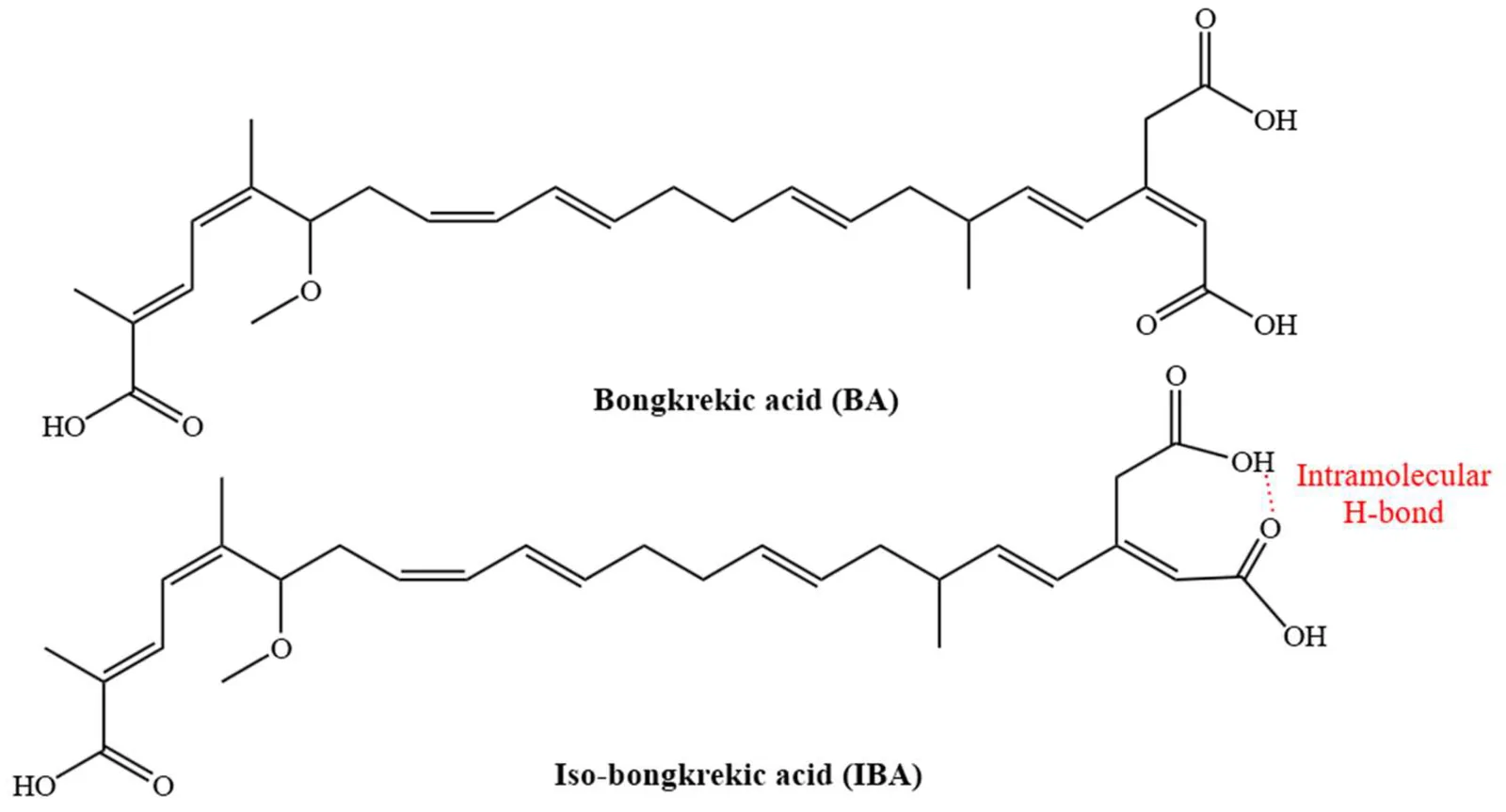
Scheme of bongkrekic acid (BA) and iso-bongkrekic acid (IBA).

**Figure 2:**
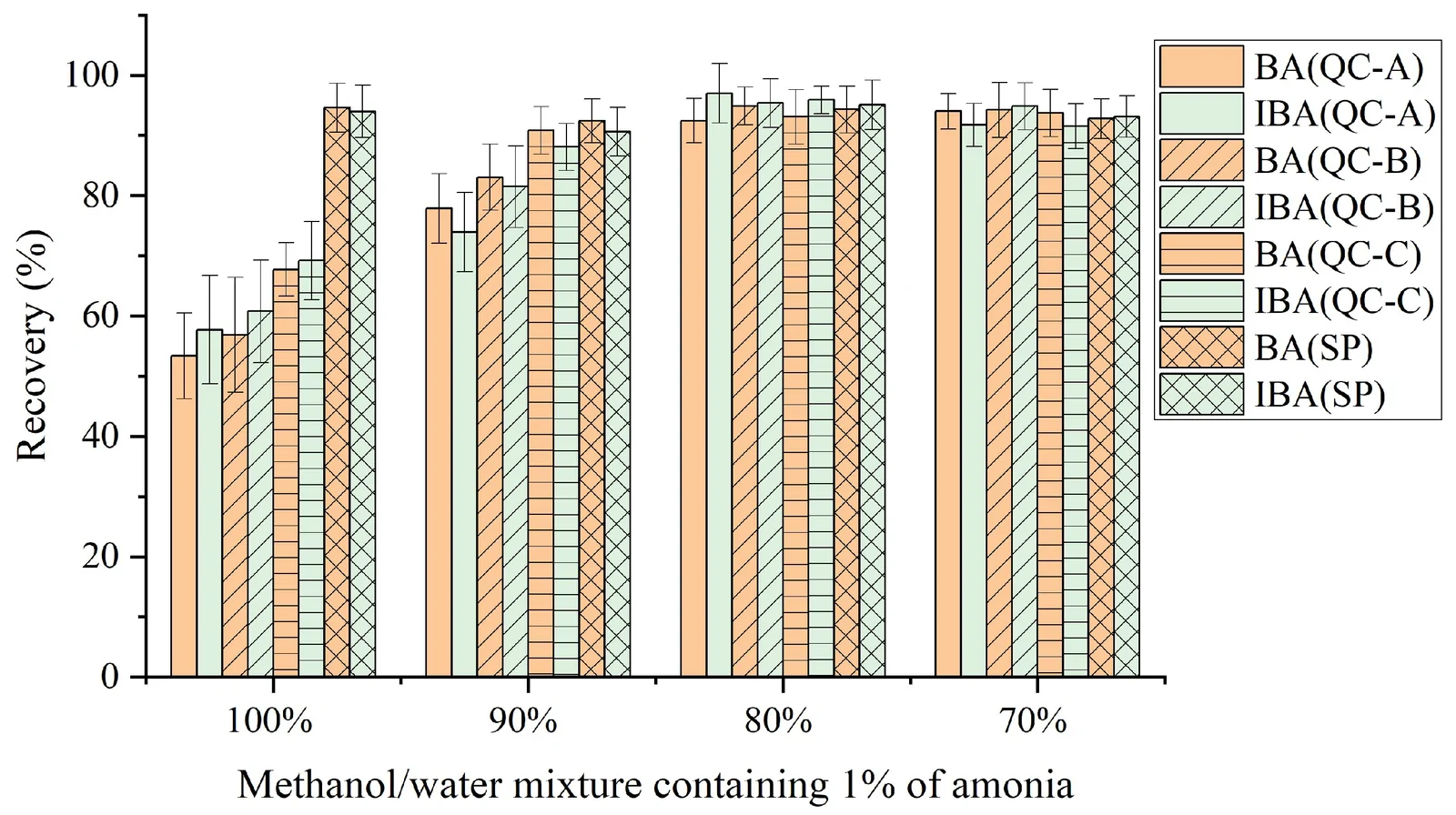
The recoveries of BA and IBA extracted from 100% to 70% methanol/water containing 1% ammonia in the spiked red yeast rice (SP), quality control (QC) samples of red yeast rice (QC-A), natto red yeast health care product (QC-B) and *monascus* red pigment (QC-C).

**Figure 3:**
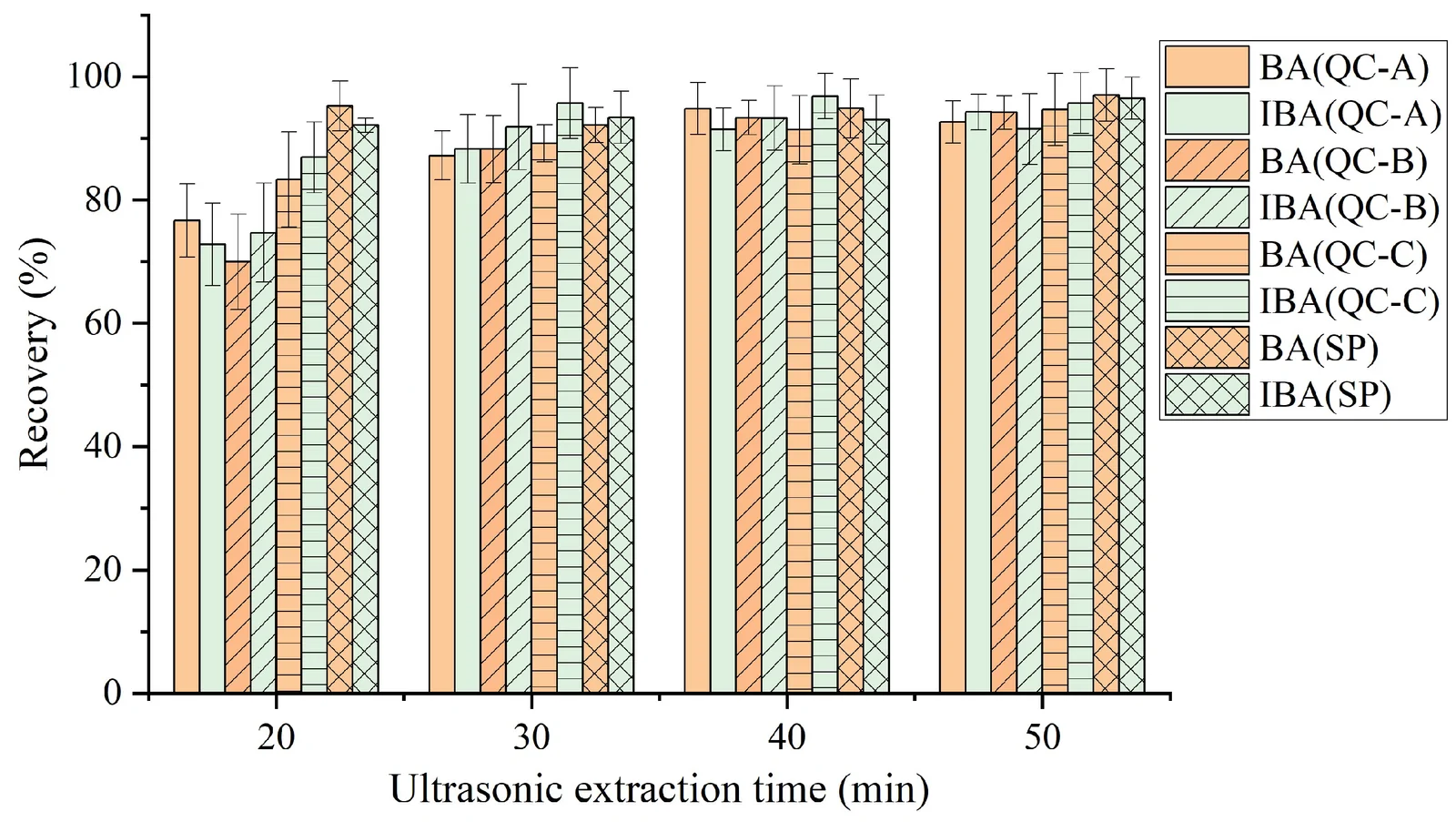
The recoveries of BA and IBA under an ultrasonic extraction time of 20 min–50 min in the spiked red yeast rice (SP), quality control (QC) samples of red yeast rice (QC-A), natto red yeast health care product (QC-B) and *monascus* red pigment (QC-C).

**Figure 4:**
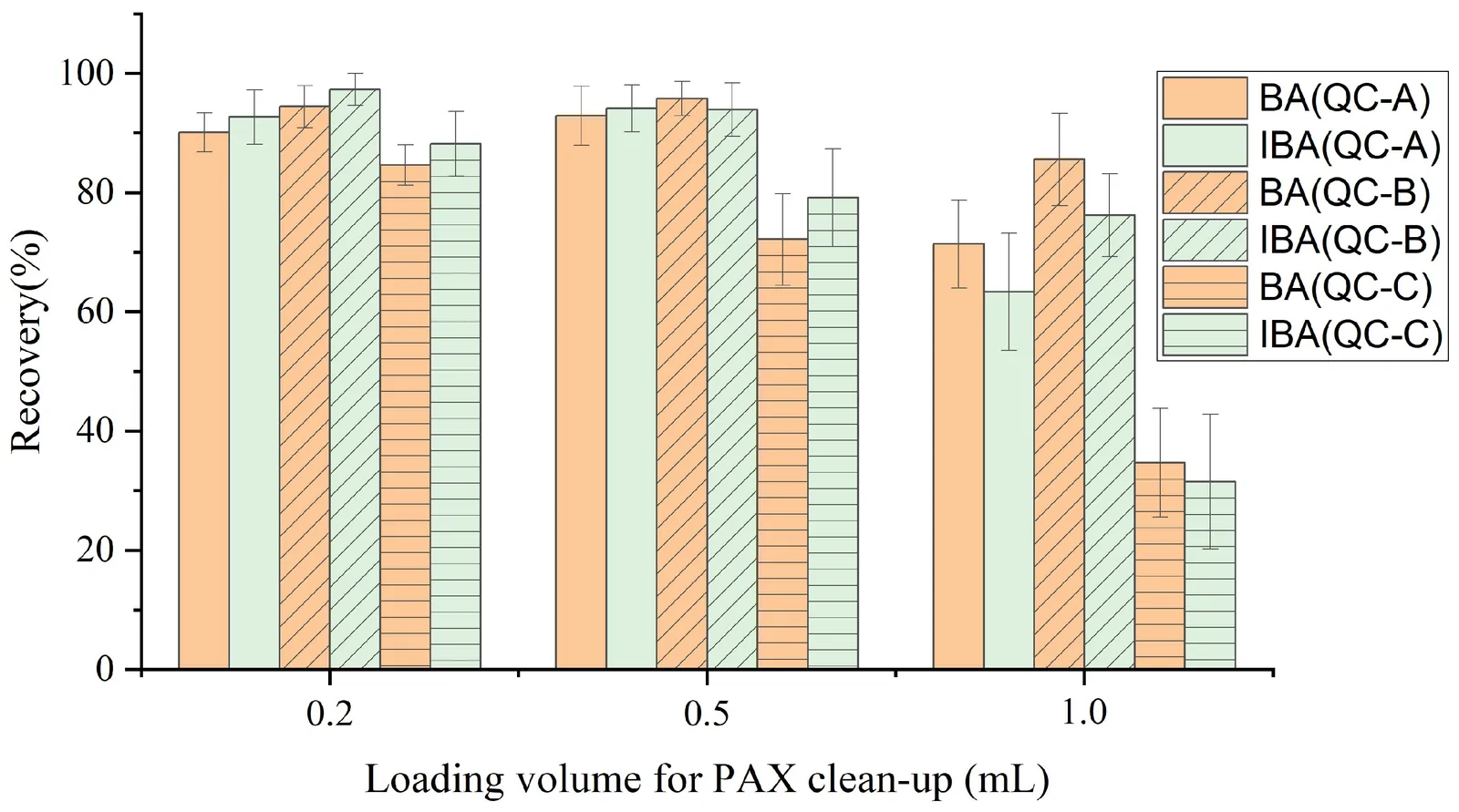
The recoveries of BA and IBA with the loading volume of 0.2 mL–1.0 mL for anion-exchange SPE clean-up in the quality control (QC) samples of red yeast rice (QC-A), natto red yeast health care product (QC-B) and *monascus* red pigment (QC-C).

**Figure 5:**
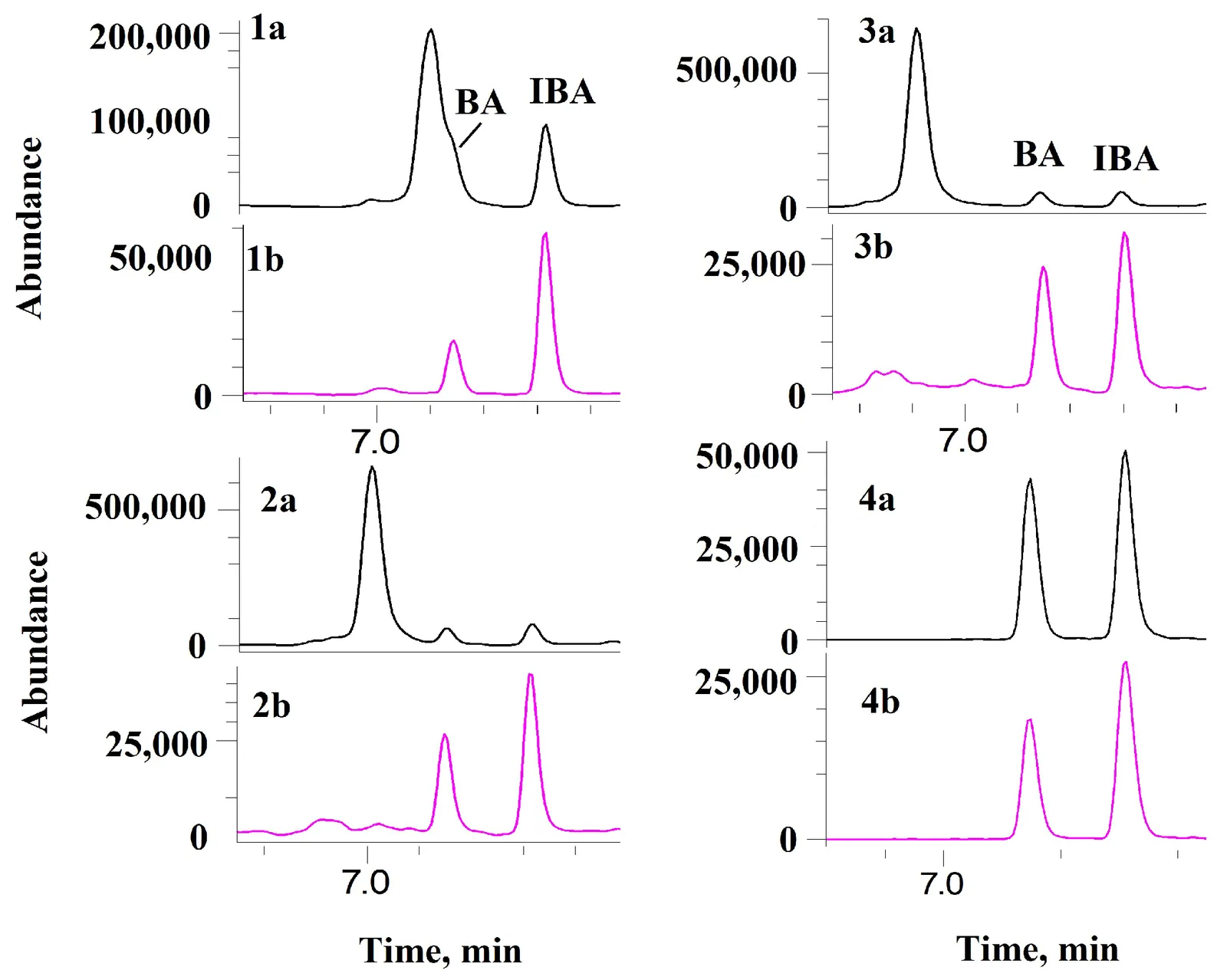
Chromatograms of BA and IBA in spiked sample and standard solution with characteristic ion channels of (a) quantitative ion (in black) and (b) qualitative ion (in pink) under different mobile phase compositions in the aqueous phase: (**1**)—spiked sample separated with 0.1% of formic acid, (**2**) spiked sample separated with 0.1% of formic acid and 0.5 mmol/L of ammonium formate, (**3**) spiked sample separated with 0.1% of formic acid and 1 mmol/L of ammonium formate, and (**4**) standard solution separated with 0.1% of formic acid and 1 mmol/L of ammonium formate.

**Figure 6:**
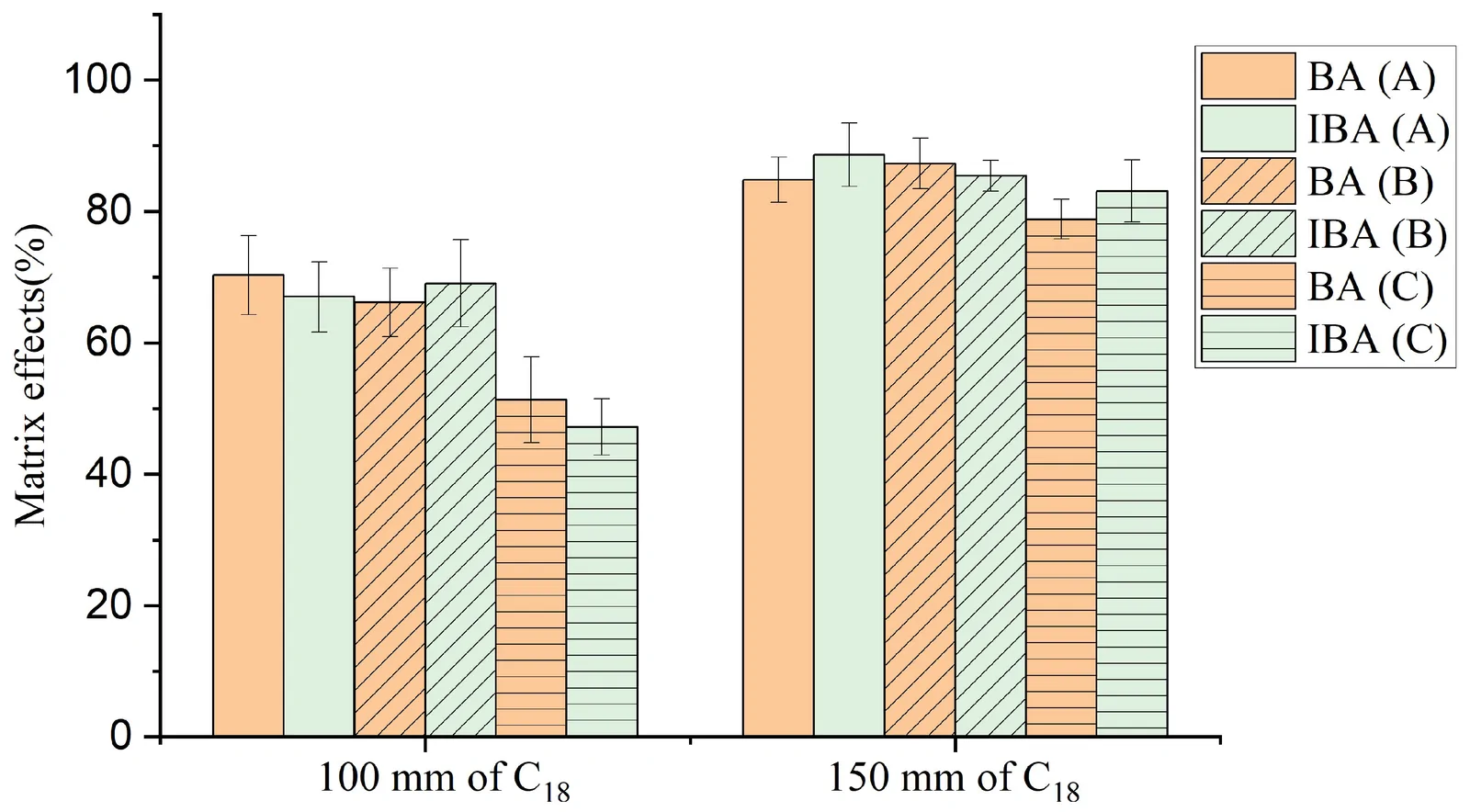
The matrix effects of BA and IBA in red yeast rice (A), natto red yeast health care product (B) and *monascus* red pigment (C) separated by C_18_ columns with lengths of 100 mm and 150 mm, respectively.

**Table 1:** Recoveries and relative standard deviations (RSDs, *n* = 6) of BA and IBA spiked in red yeast rice and its related products for intra-day accuracy and precision evaluation.

Matrix	Toxin	Spiking Level /(μg/kg)	Recovery/%						Average /%	RSD /%
			1	2	3	4	5	6		
Red yeast rice	BA	1	96.8	85.3	91.6	92.0	104	102	95.3	7.4
		20	98.8	94.7	86.8	92.3	91.2	90.3	92.3	4.4
		200	92.5	85.9	91.6	93.2	92.5	97.5	92.2	4.0
		1000	89.8	99.0	91.0	92.8	96.4	88.4	92.9	4.4
	IBA	1	109	93.7	119	98.1	114	107	107	8.9
		20	102	94.6	101	104	95.5	90.7	98.0	5.3
		200	90.7	91.6	95.0	104	88.3	85.7	92.5	6.9
		1000	94.8	102	92.4	102	95.2	96.0	97.0	4.0
Red yeast health care product	BA	1	90.9	97.2	99.0	108	94.5	92.7	97.1	6.4
		20	88.4	99.3	93.5	102	95.5	90.9	94.9	5.2
		200	94.3	91.7	89.8	94.8	90.1	96.2	92.8	2.9
		1000	92.7	91.6	101	87.2	92.8	98.1	93.9	5.2
	IBA	1	96.6	90.9	96.0	101	85.4	89.1	93.1	6.1
		20	92.1	95.4	102	98.5	90.6	92.4	95.1	4.4
		200	99.8	95.2	95.8	88.1	91.9	96.7	94.6	4.3
		1000	91.5	92.4	94.6	92.4	97.4	90.1	93.1	2.8
Monascus red pigment	BA	1.5	108	106	98.3	119	89.6	104	104	9.5
		20	92.3	103	88.5	96.4	83.9	93.7	93.0	7.0
		200	92.9	95.1	102	89.1	93.6	98.4	95.1	4.6
		1000	94.4	101	96.7	90.3	91.8	93.5	94.6	4.0
	IBA	1.5	106	109	91.6	110	81.8	91.6	98.3	11.8
		20	98.4	86.0	85.5	90.2	81.7	88.7	88.4	6.4
		200	85.3	95.1	94.4	98.2	88.9	91.6	92.2	5.0
		1000	91.2	96.9	88.9	96.2	97.2	92.0	93.7	3.7

**Table 2:** The inter-day accuracy and precision of BA and IBA in red yeast rice and its related products by testing on three consecutive days (*n* = 3).

Date	No.	Red Yeast Rice /(μg/kg)		Red Yeast Health Care Product /(μg/kg)		*Monascus* Red Pigment /(μg/kg)	
		BA	IBA	BA	IBA	BA	IBA
Day 1	1	19.2	20.7	19.8	19.5	16.5	16.8
	2	17.8	19.6	20.4	20.4	20.1	18.6
	3	17.5	19.2	17.6	19.3	17.5	21.2
Day 2	4	19.0	17.9	18.7	17.1	19.3	15.8
	5	17.7	19.0	17.6	19.9	19.7	20.1
	6	17.9	23.1	19.8	19.1	18.2	18.7
Day 3	7	22.1	18.8	17.8	16.4	15.8	20.3
	8	18.7	18.0	19.1	18.2	17.6	18.7
	9	20.0	20.1	20.7	17.5	19.2	17.9
Average content		18.9	19.6	19.0	18.6	18.2	18.7
Average accuracy/%		94.4	97.9	95.2	93.1	91.1	93.5
RSD/%		7.8	8.1	6.3	7.3	8.2	9.2

**Table 3:** The concentrations of BA and IBA in red yeast rice and its related products.

	Red Yeast Rice		Red Yeast Health Care Product		*Monascus* Red Pigments	
	BA	IBA	BA	IBA	BA	IBA
Maximum, μg/kg	17.7	12.4	9.1	7.6	1320	1647
P_97.5_, μg/kg	6.6	3.8	6.6	6.1	34.0	39.1
Average, μg/kg	0.9	0.8	1.1	1.0	50.3	54.9
Positive sample	18	13	5	4	6	6
Total sample	80	80	26	26	29	29
Detection rate, %	22.5	16.3	19.2	15.4	20.7	20.7

## Data Availability

The original contributions presented in this study are included in the article. Further inquiries can be directed to the corresponding authors.
